# Targeting sleep and the circadian system as a novel treatment strategy for Parkinson’s disease

**DOI:** 10.1007/s00415-023-12073-7

**Published:** 2023-11-09

**Authors:** Beatrix Feigl, Simon J. G. Lewis, Oliver Rawashdeh

**Affiliations:** 1 Centre for Vision and Eye Research, Queensland University of Technology (QUT), Brisbane, QLD 4059 Australia; 2 School of Biomedical Sciences, Queensland University of Technology (QUT), Brisbane, QLD 4059 Australia; 3 Queensland Eye Institute, South Brisbane, QLD 4101 Australia; 4 Parkinson’s Disease Research Clinic, Brain and Mind Centre, School of Medical Sciences, University of Sydney, Camperdown, NSW 2006 Australia; 5 School of Biomedical Sciences, Faculty of Medicine, The University of Queensland, Brisbane, QLD 4072 Australia

**Keywords:** Chronobiology, Circadian, Photoreceptor, Small molecule, Orexin, Neurodegeneration, Disease modifying Therapy

## Abstract

There is a growing appreciation of the wide range of sleep–wake disturbances that occur frequently in Parkinson’s disease. These are known to be associated with a range of motor and non-motor symptoms and significantly impact not only on the quality of life of the patient, but also on their bed partner. The underlying causes for fragmented sleep and daytime somnolence are no doubt multifactorial but there is clear evidence for circadian disruption in Parkinson’s disease. This appears to be occurring not only as a result of the neuropathological changes that occur across a distributed neural network, but even down to the cellular level. Such observations indicate that circadian changes may in fact be a driver of neurodegeneration, as well as a cause for some of the sleep–wake symptoms observed in Parkinson’s disease. Thus, efforts are now required to evaluate approaches including the prescription of precision medicine to modulate photoreceptor activation ratios that reflect daylight inputs to the circadian pacemaker, the use of small molecules to target clock genes, the manipulation of orexin pathways that could help restore the circadian system, to offer novel symptomatic and novel disease modifying strategies.

## Introduction

Therapeutic approaches in the field of Parkinson’s disease (PD) are in a significant state of flux, which is a good thing!

After discovering the positive effect of levodopa in the 1960’s, there was a steady expansion across treatment classes targeting the dopaminergic system in PD (e.g., dopamine agonists, MAOI-B, COMT-I). This was followed by the increased recognition of motor fluctuations and the evolution of surgical approaches utilising deep brain stimulation techniques to manage them. All of these advances were focused (by necessity) on the dominant motor features of the disease with little attention being directed towards the increasingly problematic plethora of non-motor symptoms that are now recognised as very much a part of PD. However, to paraphrase the Nobel Laureate, ‘*times, they are a-changin’*’. Indeed, much of the recent expenditure seen in clinical trials has been shifting towards the much welcome framework of disease modification. Whilst not yet showing any significant evidence of success, it is early days for these fledgling efforts. As such, it is probably an opportune time to widen the lens before disappearing down a ‘monoclonal’ rabbit-hole, which if anything like the experiences seen in the field of Alzheimer’s, may leave us feel as though we are tumbling helplessly like Alice, before we reach the Wonderland.

The list of potential neuroprotective targets in PD would seem to be myriad including oxidative stress, mitochondrial dysfunction, calcium homeostasis, ferroptosis and neuroinflammation [1] but one area deserving of greater attention would appear to be circadian dysfunction. Whilst there has been an increasing recognition about the potential role of the glymphatic system in the overnight clearance of amyloid in Alzheimer’s Dementia [2], little consideration has been given to the circadian processes occurring at the level of the cell.

Another area that has been largely overlooked to date is the concept of chronotherapy where the administration of medications should be timed to an individual’s circadian rhythm. This approach could also be used to achieve a better pharmacokinetic profile, improve efficacy and/or reduce toxicity related to drug metabolism. Thus, further research into the field of chronopharmacology is urgently required to explore how the body’s internal clock affects drug metabolism, efficacy and safety in PD [3].

It is increasingly understood that many neurodegenerative conditions have tight correlations with circadian dysfunction sleep disorders, most prominently in PD [4, 5]. Indeed, evidence suggests that sleep disruption is a central aspect of neurodegenerative disorder prodromes [6–8]. Circadian and sleep disruptions have significant adverse consequences on both motor and non-motor symptoms in PD, in addition to impacting on their caregivers [9, 10]. These effects are also known to carry increased cardiovascular risks in PD [11].

Complex neural connections integrate the circadian signal with the sleep homeostat, which is known to balance wake-promoting (Ascending Arousal System) and sleep-promoting (hypothalamic ventrolateral preoptic area (VLPO)) regions, as well as the ultradian system, which controls the transition between Rapid Eye Movement (REM) and Non-REM sleep stages throughout the night. Importantly, in PD neuropathological changes have been described across a number of critical brain regions in these systems that could account for sleep–wake disruption (for review see, [12]). Furthermore, there is an increasing body of evidence that suggests that a dysregulation in circadian oscillations at the cellular level may be a pathway to the accumulation of toxic waste or protein aggregation, which might in turn play a major role in neurodegeneration (for review see, [13]).

Currently, no pharmacological (e.g., Melatonin) or non-pharmacological (e.g., Light Therapy) strategies have been widely adopted to reduce the sleep disturbances in PD [14]. Furthermore, little has been done to relieve symptoms or explore any role for disease modification by targeting circadian biology. Thus, whilst appearing to be overly parsimonious, there is a clear rationale for targeting sleep and chronobiology as a novel treatment strategy for PD.

## The circadian network

The suprachiasmatic nucleus (SCN) serves as the major circadian pacemaker and is located in the anterior hypothalamus above the optic chiasm. It consists of a pair of nuclei with ~ 10,000 neurons each that have an endogenous rhythm with a period length of about 24 h in humans [15]. The neurons of the SCN are not photosensitive and photoperiodic information of light/dark stimuli is  relayed to them by retinal photoreceptors via the retinohypothalamic tract. Originally, the rods and three cone photoreceptor types located within the outer layers of the neuroretina were thought to be the only cells responsible for transmitting light information to regulate circadian rhythms. However, the textbooks are now being rewritten following the discovery of a third, inner retinal photoreceptor class, the melanopsin containing intrinsically photosensitive Retinal Ganglion Cells (ipRGCs) [16] or in short, melanopsin cells. These melanopsin cells are major contributors to non-image forming circadian processes as well as having a role in human vision [17, 18]. Melanopsin cells project via the retinohypothalamic tract to multiple brain regions including those regulating circadian rhythms, such as sleep promoting regions (via the SCN and the ventrolateral preoptic nucleus, VLPO) [19], mood (via the lateral habenulae) [20] and visual functions (via the lateral geniculate nucleus) including brightness detection and contrast vision [21, 22] (for review see [23]). In addition, melanopsin cells also form the afferent pathway of the pupillary light reflex through their projections to the olivary pretectal nucleus (OPN) that connects with the Edinger Westphal nucleus [24]. As such, the pupillary light reflex and in particular, the post-illumination pupillary light response (PIPR), which is the sustained pupil constriction after light offset, is completely driven by melanopsin [25].

The SCN signals temporal information to the pineal gland, resulting in the daytime inhibition and nighttime release of pineal melatonin [26]. Melatonin, “the hormone of darkness” has a soporific and chronobiotic function, and as the “hand” of the central clock conveys the time signals to the rest of the body, for example to initiate sleep [27]. Sleep propensity occurs approximately 2 h after melatonin secretion [28]. The relative timing of bodily processes, including that of melanopsin cell responses can be phase related to melatonin rhythm. Melanopsin function gradually decreases prior to melatonin onset and reaches a minimum after melatonin onset [29, 30]. This relationship between melanopsin and melatonin is independent of exogenous circadian cues. Hence, melanopsin cells can be considered the “crown” of the movement that sets the central clock (SCN) to the external environment to control its “hand” (melatonin).

## The role of CLOCK genes

Biological clocks are comprised of an input, a molecular timekeeping mechanism (oscillator), and physiological outputs. Input refers to cues that provide temporal information to the oscillator known as *Zeitgeber* (German for “time givers”) [31], such as light. Other clock inputs can comprise food intake, body temperature and exercise. The molecular core oscillator consists of interlocked transcriptional/translational feedback loops (TTFL) that are autonomous and self-regulating.

**Fig. 1:**
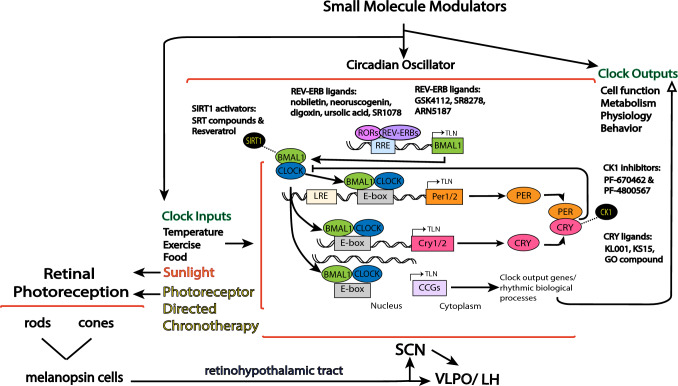
The mammalian circadian system and representative small-molecule modulators for the core (BMAL1/CLOCK/NPAS2 and PERs/CRYs) and regulatory (REV-ERBs and RORs) components of the circadian oscillator. The circadian clock consists of input pathways (e.g., retinohypothalamic tract), oscillators, and output pathways, the latter of which regulate many cellular function and behaviour to mention a few. Various small molecules have been determined to regulate core clock components or its regulatory elements. Natural daylight (e.g., sunlight) or chronotherapies targeted to retinal rod/cone and melanopsin photoreceptors activate sleep and sleep promoting regions (suprachiasmatic nucleus, SCN and ventrolateral preoptic nucleus, VLPO) and mood centers (lateral habenulae, LH) via the retinohypothalamic tract. Light can also affect the timing of circadian oscillators

Figure 1 illustrates a simplified schematic of the mammalian TTFL. Briefly, in the central TTFL, the basic helix–loop–helix transcription factors ARNTL, also known as BMAL1 brain and muscle Arnt-like protein-1 (BMAL1) and circadian locomotor output cycles kaput (CLOCK), dimerize and bind to E-boxes on promoters of *Cryptochrome* [(*Cry*)*1/2*] and *Per* (*Per1–3*) genes [32]. PER and CRY proteins dimerize in the cytoplasm before translocating into the nucleus, where they bind to and inhibit E-box transactivation by BMAL1-CLOCK, thus suppressing their own gene expression. This molecular oscillation is self-sustained and oscillates with a remarkably precise period of ∼24 h [33]. The rhythmic clock proteins in turn control output genes that affect physiological, metabolic, and behavioral rhythms [34–36], hence the coupling of physiological processes (outputs) to upstream circadian oscillators.

## Circadian network disruptions in Parkinson’s disease

Given the widespread neuropathological changes associated with PD, systemic circadian network disruptions can occur in the absence of neuropathology within the SCN. For example, intrinsically photosensitive retinal ganglion cells (ipRGCs) become dysfunctional [37, 38] and degenerate [39] in PD. The presence of Lewy bodies and Lewy neurites in the pineal gland in some individuals with PD [40] may be potentially related to neuropathology. There is also a growing appreciation of the role played by a complex system of clock genes that regulate circadian rhythm at the cellular level (e.g., cell cycle check points and cell cycle progression [41]). Thus, there is a real need for studies on the structural and functional integrity of the circadian network and other sleep wake regulators that occurs with disease progression throughout the course of α-synuclein-specific neurodegeneration. Studies are emerging that suggest these mechanisms may also be impacted by neurodegeneration or could potentially even be driving it [13]. As such, there is growing interest in the use and development of animal models and human studies for investigating sleep and circadian function in health and disease [42].

### Melanopsin dysfunction in PD

Normal structure and function of melanopsin cells, rods and cones, as well as regular melatonin secretion are required to promote the full effects of sleep [43, 44]. People with neurodegenerative and neuroretinal disease experience loss of melanopsin function [37, 45–47]. There have been several fundamental discoveries concerning the mechanism through which melanopsin photoreceptors affect circadian function and sleep in people with PD. For example, melanopsin function is impaired in people with PD and not related to any ophthalmological symptoms [38]. This functional deficit is reflected in histological examination of wholemount post-mortem retinae of eyes in PD patients that identified morphological changes to melanopsin cells [39]. The melanopsin cells are also reduced in their number and dendritic density with shorter ramifications and fewer synaptic contacts [39]. In addition, recent work has revealed that melanopsin dysfunction is correlated with reduced sleep quality and retinal thickness in PD [37]. In the same study, individuals with PD also demonstrated an abnormal dim light melatonin onset (DLMO) and an increased phase angle of entrainment compared to a healthy age-matched group [37]. Altogether, the evidence indicates that melanopsin deficits contribute to sleep and circadian disruption in PD and that they have a direct relationship with the secretion of melatonin [48].

### Pineal melatonin dysregulation in PD

While sleep and circadian rhythms change with age, it is recognised that PD patients experience much more severe sleep–wake dysfunction than typical age-related disruptions [49, 50]. Since the timing of the sleep–wake cycle is defined by the circadian system it is an accessible measurement to assess circadian disruption. The determination of (salivary or plasma) dim light melatonin onset is the gold standard for determining circadian phase [51]. Previous studies evaluating the dim light onset of melatonin secretion in PD have shown mixed results depending on disease stage and medication use. Whilst some studies have reported that patients in the early stages of PD did not demonstrate a significant phase advance (early melatonin secretion) [52], others have seen this change in medicated patients [53, 54]. Likewise mixed results have been reported regarding melatonin secretion amplitude with one study reporting increased [54] and another decreased [55] melatonin concentration in PD.

### Peripheral clock gene expression deficits in PD

The expression of clock genes within the circadian network has most frequently been investigated in animal models of dopaminergic nigrostriatal neuronal degeneration mimicking PD pathology [56]. For example, Hayashi et al. found that the expression of clock genes like *BMAL1, PERs, and CRYs* decreased in amplitude [57]. Interestingly, when investigating the potential effect of long-term treatment with levodopa on the circadian rhythm deregulation in the 6-hydroxydopamine PD mouse model, Li et al. reported that L-dopa treatment further downregulated *BMAL1* expression in the SCN and the striatum [58].

Further evidence of a relationship between PD and circadian rhythms can also be derived from the expression of clock genes in humans. For example, striking abnormalities have been found in PD patients regarding the core clock gene, *BMAL1*, including a significant lower expression in the 12-h night period compared to healthy controls, where its expression levels correlated with the severity of motor symptoms and sleep quality [59]. Other work has demonstrated a reduction in the time-dependent variation of *BMAL1* expression [52], and an association between a *BMAL1* variant and the tremor-dominant subtype of PD [60]. Furthermore, changes in the expression of the core clock genes, *PERs* and *NR1D1* have also been reported in PD [52]. Therefore, dysregulation in clock genes may influence both pathophysiology and symptomatology and a greater understanding of these relationships might allow more targeted therapies in the future.

### Rest-activity cycles in PD

Many studies have reported changes in actigraphy-based sleep–wake rhythms in individuals with PD (reviewed in [61]). These changes include parameters like an overall dampening in the amplitude of the sleep–wake rhythm and increased inter-daily variability in the timing of the rhythm onset/offset [62, 63]. However, because many other ‘indirect’ factors (e.g., medications, sleep disorders and autonomic dysfunction) can contribute to sleep–wake cycle disturbances [64], including the uncoupling of circadian and sleep regulation [54], determining the degree to which circadian disruption is responsible for dysregulating the rest-activity rhythms in PD remains a real challenge.

### Core body temperature rhythm in PD

One of the first physiological variables subjected to long-term monitoring that allowed the determination of daily rhythmicity was body temperature [65]. Indeed, the rhythmicity of core body temperature is a convenient marker of the integrity of the circadian clock network and for studies on sleep. Since the circadian rhythm interacts with the concurrent processes of cellular metabolism, core body temperature can also be measured to gauge the interaction between homeostasis and circadian rhythmicity (for reviews see [66–69]).

Previous work has indicated that overall, the 24-h rhythm of core body temperature is preserved in PD [70]. However, evidence indicates that the mesor of core body temperature is lower in individuals with PD than in healthy controls [71] and is particularly prominent in individuals with PD and coexistent depression [72]. Furthermore, the association between changes in the thermoregulatory rhythms and REM Sleep Behaviour Disorder (RBD) has recently been reported [71]. This work has identified a negative correlation between the amplitude of the overnight core body temperature and the severity of RBD symptoms [71]. However, since these changes in core body temperature were not observed in individuals with PD who did not exhibit RBD, it was concluded that the alteration in core body temperature rhythm may more likely be associated with the local brainstem pathology underlying RBD [73]. Further investigations of core body temperature rhythms will be needed to establish if circadian dysregulation is a key physiological feature, especially at the prodromal stage of synucleinopathies such as that observed with isolated RBD.

## Current circadian treatment strategies

### Photoreceptor-directed chronotherapy

Supplemental Bright Light Therapy (BLT) is a safe and established treatment for seasonal depressive disorders and depression [74] and has gained interest as a therapeutic or “photoceutical” in PD through its effects on motor and non-motor symptoms including sleep and chronobiology disturbances (for review [3]). In particular, a positive effect on daytime sleepiness [75], as well as an improvement in motor function [76–78] have been reported but recent meta-analyses deemed these results as inconclusive warranting future randomised clinical trials  [79, 80].

A major limitation of such BLT studies is that they used a variety of different fixed light spectra and intensities that are not intended to biologically effect a change in specific photoreceptor class inputs to the brain’s central circadian clock, which would be required for regular photoentrainment [81]. Indeed, such artificial lighting may even have negative health effects by delivering continuous exposure to lighting spectrums that are not specifically required, a situation akin to taking the wrong dosage of a medication. The functional and structural melanopsin changes in PD provide a strong rationale for specifically targeting the melanopsin photoreceptor using day light spectra designed to target their activity as a novel photoceutical to  better manage the clinical symptoms [3, 82, 83].

Such novel photoreceptor-directed lights can generate complex light spectra and illumination levels to match the change in the relative activity of all photoreceptors in the eye during the solar day through selectively and/or combined activation of melanopsin cells, rods and cones [83]. One recent study that has finished recruitment has specifically targeted the melanopsin deficits in PD with such biologically-directed light through a randomised, double-blind clinical trial (ACTRN12621000077864). This clinical trial has, for the first time, also addressed the individual patient’s light requirements based on their objectively measured melanopsin function and chronotype. This precision medicine approach is seen as an advance on the previous “one size fits all” adopted by light intervention studies performed to date. These are promising first steps towards targeted chronotherapy and the future may see tailored light spectrums prescribed for the therapeutic management of sleep and circadian dysfunction that address specific symptoms and potentially even disease modification.

### Targeting melatonin dysregulation

The effects of supplemental melatonin on sleep behavior in PD have been tested in several studies with mixed results, potentially due to the varying dopaminergic treatment of study participants impacting on melatonin amplitude [54]. A recent meta-analysis and systematic review of 7 studies concluded that melatonin significantly improved objective (total sleep time as assessed with polysomnography) and subjective sleep quality (assessed with the Pittsburgh Sleep Quality Index questionnaire) [84]. However, supplemental melatonin is not effective in decreasing excessive daytime sleepiness and rapid eye movement sleep behaviour disorder (RBD), two commonly experienced sleep disturbances in PD [7, 84, 85]. An evidence-based review on the treatment of non-motor symptoms in PD concluded that although there is insufficient evidence for melatonin to be beneficial for treatment of insomnia, it is regarded as “possibly useful” [14].

### Exercise- and fasting-directed chronotherapy approaches

Prescribed exercise is another circadian-based strategy that has been developed to restore circadian function. Whilst proof-of-concept studies were originally developed in rodent models [86, 87], they have since been translated into the PD population showing both improvements in objective and subjective sleep measurements [88–90].

Intermittent fasting (IF) has been shown to be protective against nigral dopaminergic neurons from MPTP-mediated dopaminergic neuronal injury in mice and neuronal excitotoxicity in rats and mice [91]. A 6-month study of caloric restriction in a primate PD model led to better locomotor activity with higher striatal dopamine levels relative to ad libitum-fed controls [92]. Similarly, Griffioen and colleagues showed that IF led to a decreased burden of alpha-synuclein in the brainstem that contributes to autonomic dysfunction commonly seen in PD [93].

The circadian clock intimately interacts with nutrient-sensing pathways, allowing feeding-fasting rhythms to enhance the robustness of the oscillation of circadian activator and repressor components that bind to the transcriptional regulatory regions of thousands of genes, which in turn generate rhythms in metabolism, cell division and repair, and growth [91, 94–100]. Accordingly, fasting-associated interventions may be targeting mitochondrial dysfunction and its downstream consequences rather than acting as a nutritional supplement, as it likely targets several physiological pathways. Thus fasting-associated interventions would appear feasible, effective, and inexpensive circadian-based treatments that are currently being tested in preclinical and clinical settings [94–96, 101, 102].

## Potential circadian treatment strategies

### Modulating the cellular clock

Small molecule drugs are chemically synthesised compounds with a molecular weight commonly below ~ 500 Da [103, 104]. Recent work has highlighted that novel “small molecules” can manipulate the circadian clock either directly by acting on the oscillator (e.g., binding to core clock proteins to modulate clock protein–protein interaction) or through key regulators (e.g., clock-associated cellular pathways involving ubiquitinases, kinases and phosphatases [105, 106]), via input pathways, or feedback mechanisms from output targets (e.g., pineal melatonin and adrenal glucocorticoids), see Fig. 1. Clock-modulating small molecules could directly manipulate the circadian system to improve clock-regulated output processes (e.g., cognition), alleviate disease symptoms and pathological decline [107–111]. Indeed, recent work has revealed that casein kinase 1 (CK1) ε/δ, a key regulator of the circadian oscillator, may be a suitable target for the therapeutic intervention against the cognitive decline in AD [112]. Whether this target is specific for AD or could apply to PD dementia requires further consideration.

### Targeting clock outputs

An alternative strategy for normalising circadian disturbance would be to target the downstream outputs of the circadian clock (e.g., orexinergic neurons) [113]. The discovery of the neuropeptide orexin in 1998 [114, 115] has triggered enormous research efforts looking for druggable targets. Orexin is a key modulator of the sleep–wake cycle [116] and the orexin system also has projections to brain regions that have been implicated in arousal and cognition [117]. Studies conducted in PD have reported pathological changes in the lateral hypothalamus with a loss of orexin neurons and fluctuations in CSF orexin levels [118]. More recent work has also suggested the potential protective action and therapeutic applications of orexin receptor agonists in preclinical models of PD [119], as well as highlighting the possibility that orexin receptor antagonists may consolidate the abnormal sleep patterns observed in PD [120].

Both dual orexin receptor antagonists (DORAs) and selective orexin receptor antagonists (SORAs) have recently been developed for the short-term and long-term treatment of insomnia, aiming for fewer side effects than existing hypnotic drugs [121]. Since orexin neuron activation is under pronounced circadian control, it is possible that DORAs and SORAs may need to have specifically timed administration, based on circadian rhythms. Studies conducted on the assessment of sleep architecture in patients with insomnia, major depressive disorders, and obstructive sleep apnoea have shown that DORAs increase the total sleep time by promoting REM sleep, without affecting, or at the expense of decreasing, non-REM sleep [122]. Thus, such agents might potentially offer an approach for treating the broad spectrum of sleep disorders in PD and further specific trials are required in this patient population.

## Conclusion

It is clear that modulating photoreceptor activation ratios that reflect daylight inputs to the SCN and the retinal pathologies found in PD may present opportunities to develop mechanism based light therapy protocols [3], such as using light spectra tailored for new photoceutical treatments to better manage the clinical symptoms in PD [83]. In line with the importance of the timing of drugs, it is likely that these targeted light therapy protocols would need to be adjusted to synchronise with melatonin action (to fall asleep and maintain sleep) and the timing of other medications.

Utilising chronotherapies and photoceuticals to restore normal cellular processes to improve the sequestration and elimination of misfolded proteins may also act to slow neurodegenerative disease progression. Indeed, there is a growing support that not only is circadian and sleep dysfunction a consequence of neurodegeneration, but may also play a causative role, predisposing to disease onset and exacerbating disease progression. In this scenario, circadian dysfunction and neurodegeneration would form a detrimental, self-perpetuating positive-feedback loop (reviewed in [4, 5, 123–125]). It is clear that the sleep–wake cycle and circadian facets of disease are of great importance when examining major neurodegenerative diseases and represent novel targets for treatment (for review see [3, 61, 126]). Thus, considering that many aspects of life from cellular functions to physiology and behaviour are circadian regulated, restoration or normalisation of this disruption in PD may offer a range of therapeutic targets for both symptomatic and disease modifying therapies.

## References

[1] Lewis SJG (2018). Disease-modifying approaches for Parkinson disease. Med J Aust.

[2] Tarasoff-Conway JM (2015). Clearance systems in the brain-implications for Alzheimer disease. Nat Rev Neurol.

[3] Fifel K, Videnovic A (2019). Chronotherapies for Parkinson's disease. Prog Neurobiol.

[4] Mattis J, Sehgal A (2016). Circadian rhythms, sleep, and disorders of aging. Trends Endocrinol Metab.

[5] Leng Y (2019). Association between circadian rhythms and neurodegenerative diseases. Lancet Neurol.

[6] Musiek ES (2018). Circadian rest-activity pattern changes in aging and preclinical Alzheimer disease. JAMA Neurol.

[7] Leng Y (2018). Excessive daytime sleepiness, objective napping and 11-year risk of Parkinson's disease in older men. Int J Epidemiol.

[8] Lazar AS (2015). Sleep deficits but no metabolic deficits in premanifest Huntington's disease. Ann Neurol.

[9] Naismith SL (2011). Neuropsychological functioning in Parkinson's disease: differential relationships with self-reported sleep-wake disturbances. Mov Disord.

[10] Naismith SL, Hickie IB, Lewis SJG (2010). The role of mild depression in sleep disturbance and quality of life in Parkinson’s disease. J Neuropsychiatry Clin Neurosci.

[11] Berganzo K (2013). Nocturnal hypertension and dysautonomia in patients with Parkinson's disease: are they related?. J Neurol.

[12] Zhong G (2011). Sleep-wake disturbances in common neurodegenerative diseases: a closer look at selected aspects of the neural circuitry. J Neurol Sci.

[13] Nassan M, Videnovic A (2022). Circadian rhythms in neurodegenerative disorders. Nat Rev Neurol.

[14] Seppi K (2019). Update on treatments for nonmotor symptoms of Parkinson's disease-an evidence-based medicine review. Mov Disord.

[15] Czeisler CA (1999). Stability, precision, and near-24-hour period of the human circadian pacemaker. Science.

[16] Provencio I (2000). A novel human opsin in the inner retina. J Neurosci.

[17] Dacey DM (2005). Melanopsin-expressing ganglion cells in primate retina signal colour and irradiance and project to the LGN. Nature.

[18] Hattar S (2006). Central projections of melanopsin expressing retinal ganglion cells in the mouse. J Comp Neurol.

[19] Provencio I (1998). Melanopsin: An opsin in melanophores, brain, and eye. Proc Natl Acad Sci U S A.

[20] LeGates TA, Fernandez DC, Hattar S (2014). Light as a central modulator of circadian rhythms, sleep and affect. Nat Rev Neurosci.

[21] Zele AJ (2018). Cone and melanopsin contributions to human brightness estimation. JOSA.

[22] Uprety Samir (2022). Rhodopsin and melanopsin contributions to human brightness estimation. iScience.

[23] Joyce DS (2023). Melanopsin vision.

[24] Gamlin PDR (2007). Human and macaque pupil responses driven by melanopsin-containing retinal ganglion cells. Vision Res.

[25] Kelbsch C (2019). Standards in Pupillography. Front Neurol.

[26] Sack RL, Lewy AJ, Hughes RJ (1998). Use of melatonin for sleep and circadian rhythm disorders. Ann Med.

[27] Arendt J (2000). Melatonin, circadian rhythms, and sleep. N Engl J Med.

[28] Lavie P (2001). Sleep-wake as a biological rhythm. Annu Rev Psychol.

[29] Zele AJ (2011). The circadian response of intrinsically photosensitive retinal ganglion cells. PLoS ONE.

[30] Munch M (2012). Circadian and wake-dependent effects on the pupil light reflex in response to narrow-bandwidth light pulses. Invest Ophthalmol Vis Sci.

[31] Moore RY (1997). Circadian rhythms: basic neurobiology and clinical applications. Annu Rev Med.

[32] Zhang EE, Kay SA (2010). Clocks not winding down: unravelling circadian networks. Nat Rev Mol Cell Biol.

[33] Brown SA, Schibler U (1999). The ins and outs of circadian timekeeping. Curr Opin Genet Dev.

[34] Bass J, Takahashi JS (2010). Circadian integration of metabolism and energetics. Science.

[35] Kim P (2019). Coupling the circadian clock to homeostasis: the role of period in timing physiology. Endocr Rev.

[36] Rawashdeh O (2016). Period1 gates the circadian modulation of memory-relevant signaling in mouse hippocampus by regulating the nuclear shuttling of the CREB kinase pP90RSK. J Neurochem.

[37] Feigl B (2020). Melanopsin cell dysfunction is involved in sleep disruption in Parkinson's disease. J Parkinsons Dis.

[38] Joyce DS (2018). Melanopsin-mediated pupil function is impaired in Parkinson's disease. Sci Rep.

[39] Ortuno-Lizaran I (2018). Degeneration of human photosensitive retinal ganglion cells may explain sleep and circadian rhythms disorders in Parkinson's disease. Acta Neuropathol Commun.

[40] De Pablo-Fernandez E (2018). A histologic study of the circadian system in parkinson disease, multiple system atrophy, and progressive supranuclear palsy. JAMA Neurol.

[41] Hunt T, Sassone-Corsi P (2007). Riding tandem: circadian clocks and the cell cycle. Cell.

[42] Hunt J (2022). Sleep and circadian rhythms in Parkinson's disease and preclinical models. Mol Neurodegener.

[43] Altimus CM (2008). Rods-cones and melanopsin detect light and dark to modulate sleep independent of image formation. Proc Natl Acad Sci U S A.

[44] Lack LC, Wright HR (2007). Chronobiology of sleep in humans. Cell Mol Life Sci.

[45] La Morgia C (2016). Melanopsin retinal ganglion cell loss in Alzheimer disease. Ann Neurol.

[46] Flight risk (2012) Nature 483(7390):373–374 10.1038/483373b 22437570

[47] Feigl B, Zele AJ (2014). Melanopsin-expressing intrinsically photosensitive retinal ganglion cells in retinal disease. Optom Vision Sci.

[48] Altimus CM (2010). Rod photoreceptors drive circadian photoentrainment across a wide range of light intensities. Nat Neurosci.

[49] Weissova K (2016). Moderate changes in the circadian system of Alzheimer's disease patients detected in their home environment. PLoS ONE.

[50] Videnovic A (2014). 'The clocks that time us'–circadian rhythms in neurodegenerative disorders. Nat Rev Neurol.

[51] Pandi-Perumal SR (2007). Dim light melatonin onset (DLMO): a tool for the analysis of circadian phase in human sleep and chronobiological disorders. Prog Neuropsychopharmacol Biol Psychiatry.

[52] Breen DP (2014). Sleep and circadian rhythm regulation in early Parkinson disease. JAMA Neurol.

[53] Fertl E (1991). Circadian secretion pattern of melatonin in Parkinson's disease. J Neural Transm Park Dis Dement Sect.

[54] Bolitho SJ (2014). Disturbances in melatonin secretion and circadian sleep-wake regulation in Parkinson disease. Sleep Med.

[55] Bordet R (2003). Study of circadian melatonin secretion pattern at different stages of Parkinson's disease. Clin Neuropharmacol.

[56] Chia SJ, Tan EK, Chao YX (2020). Historical perspective: models of Parkinson’s disease. Int J Mol Sci.

[57] Hayashi A (2013). A disruption mechanism of the molecular clock in a MPTP mouse model of Parkinson's disease. Neuromolecular Med.

[58] Li SY (2017). Long-term levodopa treatment accelerates the circadian rhythm dysfunction in a 6-hydroxydopamine rat model of Parkinson's disease. Chin Med J (Engl).

[59] Cai Y (2010). Expression of clock genes Per1 and Bmal1 in total leukocytes in health and Parkinson's disease. Eur J Neurol.

[60] Gu Z (2015). Association of ARNTL and PER1 genes with Parkinson's disease: a case-control study of Han Chinese. Sci Rep.

[61] Videnovic A, Willis GL (2016). Circadian system—a novel diagnostic and therapeutic target in Parkinson's disease?. Mov Disord.

[62] Obayashi K (2021). Circadian activity rhythm in Parkinson's disease: findings from the PHASE study. Sleep Med.

[63] Van Someren EJ (1997). Actigraphic monitoring of movement and rest-activity rhythms in aging, Alzheimer's disease, and Parkinson's disease. IEEE Trans Rehabil Eng.

[64] Gros P, Videnovic A (2020). Overview of sleep and circadian rhythm disorders in Parkinson disease. Clin Geriatr Med.

[65] Chossat C (1843). Recherches expérimentales sur l’inanition. III. De l’alimentation insuffisante. Ann Sci Nat Zool.

[66] Refinetti R, Menaker M (1992). The circadian rhythm of body temperature. Physiol Behav.

[67] Refinetti R (2010). The circadian rhythm of body temperature. Front Biosci (Landmark Ed).

[68] Piccione G, Refinetti R (2003). Thermal chronobiology of domestic animals. Front Biosci.

[69] Waterhouse J (2005). The circadian rhythm of core temperature: origin and some implications for exercise performance. Chronobiol Int.

[70] Pierangeli G (2001). Nocturnal body core temperature falls in Parkinson's disease but not in multiple-system atrophy. Mov Disord.

[71] Zhong G (2013). The relationship between thermoregulation and REM sleep behaviour disorder in Parkinson's disease. PLoS ONE.

[72] Suzuki K (2007). Circadian variation of core body temperature in Parkinson disease patients with depression: a potential biological marker for depression in Parkinson disease. Neuropsychobiology.

[73] Raupach AK (2020). Assessing the role of nocturnal core body temperature dysregulation as a biomarker of neurodegeneration. J Sleep Res.

[74] Golden RN (2005). The efficacy of light therapy in the treatment of mood disorders: a review and meta-analysis of the evidence. Am J Psychiatry.

[75] Videnovic A (2017). timed light therapy for sleep and daytime sleepiness associated with Parkinson disease: a randomized clinical trial. JAMA Neurol.

[76] Paus S (2007). Bright light therapy in Parkinson's disease: a pilot study. Mov Disord.

[77] Willis GL, Turner EJ (2007). Primary and secondary features of Parkinson's disease improve with strategic exposure to bright light: a case series study. Chronobiol Int.

[78] Artemenko AR, Ia Levin I (1996). The phototherapy of parkinsonism patients. Zh Nevrol Psikhiatr Im S S Korsakova.

[79] Huang HT, Huang TW, Hong CT (2021). Bright light therapy for Parkinson disease: a literature review and meta-analysis of randomized controlled trials. Biology (Basel).

[80] Lin F (2021). The effects of bright light therapy on depression and sleep disturbances in patients with Parkinson's disease: a systematic review and meta-analysis of randomized controlled trials. Sleep Med.

[81] Altimus CM (2008). Rods-cones and melanopsin detect light and dark to modulate sleep independent of image formation. Proc Natl Acad Sci USA.

[82] Roccaro I, Smirni D (2020). Fiat lux: the light became therapy. An overview on the bright light therapy in alzheimer's disease sleep disorders. J Alzheimers Dis.

[83] Feigl B, Carter D, Zele AJ (2023). Photoreceptor enhanced light therapy (PELT): a framework for implementing biologically directed integrative lighting. LEUKOS.

[84] Ma H (2022). Melatonin treatment for sleep disorders in Parkinson's disease: a meta-analysis and systematic review. Front Aging Neurosci.

[85] Gilat M (2020). Melatonin for rapid eye movement sleep behavior disorder in Parkinson's disease: a randomised controlled trial. Mov Disord.

[86] Palasz E (2019). Exercise-induced neuroprotection and recovery of motor function in animal models of parkinson's disease. Front Neurol.

[87] Leise TL (2013). Voluntary exercise can strengthen the circadian system in aged mice. Age (Dordr).

[88] Amara AW (2020). Randomized, controlled trial of exercise on objective and subjective sleep in parkinson's disease. Mov Disord.

[89] Frange C (2020). Exercise for "sleep rehabilitation" in Parkinson's disease. Mov Disord.

[90] Cristini J (2021). The effects of exercise on sleep quality in persons with Parkinson's disease: a systematic review with meta-analysis. Sleep Med Rev.

[91] Mattson MP (2018). Intermittent metabolic switching, neuroplasticity and brain health. Nat Rev Neurosci.

[92] Maswood N (2004). Caloric restriction increases neurotrophic factor levels and attenuates neurochemical and behavioral deficits in a primate model of Parkinson's disease. Proc Natl Acad Sci U S A.

[93] Griffioen KJ (2013). Dietary energy intake modifies brainstem autonomic dysfunction caused by mutant alpha-synuclein. Neurobiol Aging.

[94] Curtis WM (2022). NADPH and mitochondrial quality control as targets for a circadian-based fasting and exercise therapy for the treatment of Parkinson’s disease. Cells.

[95] Mattson MP (2014). Interventions that improve body and brain bioenergetics for Parkinson's disease risk reduction and therapy. J Parkinsons Dis.

[96] Anson RM (2003). Intermittent fasting dissociates beneficial effects of dietary restriction on glucose metabolism and neuronal resistance to injury from calorie intake. Proc Natl Acad Sci U S A.

[97] Koike N (2012). Transcriptional architecture and chromatin landscape of the core circadian clock in mammals. Science.

[98] Vollmers C (2009). Time of feeding and the intrinsic circadian clock drive rhythms in hepatic gene expression. Proc Natl Acad Sci U S A.

[99] Mohawk JA, Green CB, Takahashi JS (2012). Central and peripheral circadian clocks in mammals. Annu Rev Neurosci.

[100] Longo VD, Panda S (2016). Fasting, circadian rhythms, and time-restricted feeding in healthy lifespan. Cell Metab.

[101] Ojha U (2023). Intermittent fasting protects the nigral dopaminergic neurons from MPTP-mediated dopaminergic neuronal injury in mice. J Nutr Biochem.

[102] Neth BJ (2021). The role of intermittent fasting in Parkinson's disease. Front Neurol.

[103] Veber DF (2002). Molecular properties that influence the oral bioavailability of drug candidates. J Med Chem.

[104] Lipinski CA (2001). Experimental and computational approaches to estimate solubility and permeability in drug discovery and development settings. Adv Drug Deliv Rev.

[105] Reischl S, Kramer A (2011). Kinases and phosphatases in the mammalian circadian clock. FEBS Lett.

[106] Hirota T (2012). Identification of small molecule activators of cryptochrome. Science.

[107] Schroeder AM, Colwell CS (2013). How to fix a broken clock. Trends Pharmacol Sci.

[108] Wallach T, Kramer A (2015). Chemical chronobiology: Toward drugs manipulating time. FEBS Lett.

[109] Nohara K, Yoo SH, Chen ZJ (2015). Manipulating the circadian and sleep cycles to protect against metabolic disease. Front Endocrinol (Lausanne).

[110] Chen Z, Yoo SH, Takahashi JS (2013). Small molecule modifiers of circadian clocks. Cell Mol Life Sci.

[111] Flajolet M (2007). Regulation of Alzheimer's disease amyloid-beta formation by casein kinase I. Proc Natl Acad Sci U S A.

[112] Sundaram S (2019). Inhibition of casein kinase 1delta/epsilonimproves cognitive-affective behavior and reduces amyloid load in the APP-PS1 mouse model of Alzheimer's disease. Sci Rep.

[113] Zhang R (2014). A circadian gene expression atlas in mammals: implications for biology and medicine. Proc Natl Acad Sci U S A.

[114] Sakurai T (1998). Orexins and orexin receptors: a family of hypothalamic neuropeptides and G protein-coupled receptors that regulate feeding behavior. Cell.

[115] de Lecea L (1998). The hypocretins: hypothalamus-specific peptides with neuroexcitatory activity. Proc Natl Acad Sci U S A.

[116] Sakurai T (2007). The neural circuit of orexin (hypocretin): maintaining sleep and wakefulness. Nat Rev Neurosci.

[117] Peyron C (1998). Neurons containing hypocretin (orexin) project to multiple neuronal systems. J Neurosci.

[118] Liu C (2020). Orexin and Parkinson's disease: a protective neuropeptide with therapeutic potential. Neurochem Int.

[119] Liu MF (2018). Orexin-a exerts neuroprotective effects via OX1R in Parkinson's disease. Front Neurosci.

[120] Kumar S (2021). Exploring the role of orexinergic neurons in Parkinson's disease. Neurotox Res.

[121] Kumar A, Chanana P, Choudhary S (2016). Emerging role of orexin antagonists in insomnia therapeutics: an update on SORAs and DORAs. Pharmacol Rep.

[122] Clark JW (2020). Effects of orexin receptor antagonism on human sleep architecture: a systematic review. Sleep Med Rev.

[123] Hood S, Amir S (2017). Neurodegeneration and the circadian clock. Front Aging Neurosci.

[124] Abbott SM, Videnovic A (2016). Chronic sleep disturbance and neural injury: links to neurodegenerative disease. Nat Sci Sleep.

[125] Malhotra RK (2018). Neurodegenerative disorders and sleep. Sleep Med Clin.

[126] Amara AW, Chahine LM, Videnovic A (2017). Treatment of sleep dysfunction in Parkinson's disease. Curr Treat Options Neurol.

